# A new limit for blood metabolite analysis using ^1^H NMR spectroscopy

**DOI:** 10.1016/j.jmro.2022.100082

**Published:** 2022-11-15

**Authors:** G.A. Nagana Gowda, Vadim Pascua, Daniel Raftery

**Affiliations:** a Northwest Metabolomics Research Center; b Mitochondria Metabolism Center, Anesthesiology and Pain Medicine, University of Washington, Seattle, WA 98109, United States of America; c Fred Hutchinson Cancer Research Center, Seattle, WA 98109, United States of America

**Keywords:** Blood metabolomics, Labile metabolites, Unknown metabolite identification, 1d nmr, 2d nmr, Redox coenzymes, Energy coenzymes

## Abstract

Human blood is the most widely used biospecimen in the clinic and the metabolomics field. While both mass spectrometry and NMR spectroscopy are the two premier analytical platforms in the metabolomics field, NMR exhibits several unsurpassed characteristics for blood metabolite analysis, the most important of which are its ability to identify unknown metabolites and its quantitative nature. However, the relatively small number of metabolites accessible by NMR has restricted the scope of its applications. Enhancing the limit of identified metabolites in blood will therefore greatly impact NMR-based metabolomics. Continuing our efforts to address this major issue, our current study describes the identification of 12 metabolites, which expands the number of quantifiable blood metabolites by ~15%. These results, in combination with our earlier efforts, now provide access to nearly 90 metabolites, which is the highest to date for a simple 1D ^1^H NMR experiment that is widely used in the metabolomics field. Metabolites were identified based on the comprehensive investigation of human blood and plasma using 1D/2D NMR techniques. The newly identified metabolites were validated based on chemical shift databases, spectra of authentic compounds obtained under conditions identical to blood/plasma, and, finally, spiking experiments using authentic compounds. Considering the high reproducibility of NMR and the sensitivity of chemical shifts to altered sample conditions, experimental protocols and peak annotations are provided for the newly identified metabolites, which serve as a template for identification of blood metabolites for routine applications. Separately, the identified metabolites were evaluated for their sensitivity to preanalytical conditions. The results reveal that among the newly identified metabolites, inosine monophosphate (IMP) and nicotinamide are associated with labile coenzymes and their levels are sensitive to preanalytical conditions. The study demonstrates the expansion of quantifiable blood metabolites using NMR to a new height and is expected to greatly impact blood metabolomics.

## 1. Introduction

Blood is the most widely used biospecimen for clinical investigations as well as in the metabolomics field [1–4]. It is the primary source of biomarkers of health and virtually all diseases. Its close association with essentially every living cell in the human body combined with the relatively easy access for investigations makes it clinically more relevant when compared to other biospecimens such as urine, saliva, or biopsied tissue. Blood metabolomics is focused on the search for biomarkers of health and diseases, identifying drug targets, and understanding disease mechanisms. Mass spectrometry (MS) and NMR spectroscopy are the two major analytical platforms widely used for the analysis of blood metabolites in metabolomics. MS is a highly sensitive method and detects several hundred to thousands of metabolites, including many lipid molecules, in a single step. NMR spectroscopy, on the hand, is highly reproducible and quantitative, which are unparalleled characteristics for metabolite analysis. However, NMR is far less sensitive than MS and generally provides somewhat poorly resolved spectra for the widely used ^1^H NMR. Unlike MS, NMR generally does not utilize chromatography to separate metabolites, owing to technical challenges related to reduced sensitivity. Hence, the combination of poor sensitivity and resolution has so far resulted in far fewer metabolites that can be measured by NMR in blood and has thus restricted the scope of NMR-based blood metabolomics. New efforts to enhance the limit of identified metabolites will therefore greatly impact the blood metabolomics field.

Several efforts to date have focused on improving blood serum/plasma metabolite detection. A large amount of protein (6–8 g/dL) present in blood restricts the number of detected small molecule metabolites and also deleteriously affects the accuracy of their analysis due to protein binding [5–7]. To address this challenge, numerous efforts were focused on removing proteins from blood serum/plasma prior to analysis. These involved physically removing protein using ultra-filtration, solid phase extraction, or protein precipitation using an organic solvent such as methanol, acetonitrile, acetone, perchloric acid, or trichloroacetic acid. Such efforts have helped to increase the number of metabolite identifications to about 50 [8–13].

Subsequently, during the last several years, major efforts in our laboratory were focused on further expanding the limits of blood metabolite analysis using NMR. We optimized protein removal methods and, based on the comprehensive analysis using 1D/2D NMR techniques, established the identities of a large number of unknown metabolites (at least by NMR measurements) in blood serum and plasma [14, 15]. These efforts led to an expansion of the number of quantifiable blood metabolites to nearly 70 [15]. Subsequently, the further development of methods extended blood metabolites to cover many additional, previously unidentified, metabolites that are fundamental to the functioning of all living cells [16, 17]; these include the redox coenzymes, energy coenzymes, and antioxidants. Following these efforts, the number of identified blood metabolites using the ^1^H NMR 1D spectrum increased to nearly 80 [16, 17].

Continuing this work to further expand the limits of blood metabolite analysis using ^1^H NMR, we further expanded the number of quantifiable blood metabolites in the present study by ~15%. These results combined with our earlier investigations [15–17] now enable access to nearly 90 identified blood metabolites, which is the largest number thus far for blood metabolites detected by a simple 1D NMR experiment. As with our previous studies, we have provided peak annotations for the characteristic peaks from the newly identified metabolites in the NMR spectrum. This is essential for NMR-based blood metabolomics since peak identification using chemical shift databases and software tools is often inadequate for reliable identification of metabolites owing to the narrow dispersion of peaks and the sensitivity of NMR peaks to the sample preparation and analysis conditions. This study represents another major step for ^1^H NMR-based blood metabolomics and promises to lead to additional applications and metabolomics studies of health and disease.

## 2. Materials and methods

### 2.1. Chemicals and solvents

Methanol, chloroform, sodium phosphate (monobasic; NaH_2_PO_4_), sodium phosphate (dibasic; Na_2_HPO_4_), and 3-(trimethylsilyl)propionic acid-2,2,3,3-d_4_ sodium salt (TSP) were obtained from Sigma-Aldrich (St. Louis, MO). Standard compounds used to obtain spectra under conditions identical to blood and plasma and for spiking experiments to confirm assignments were obtained from Sigma-Aldrich (St. Louis, MO). Deuterium oxide (D_2_O) was obtained from Cambridge Isotope Laboratories, Inc. (Andover, MA). Deionized (DI) water was purified using an in-house Synergy Ultrapure Water System from Millipore (Billerica, MA). All chemicals were used with no further purification.

### 2.2. Biospecimens

Whole blood and plasma from the same (deidentified) individuals were purchased from Solomon Park Research Laboratories, Inc. (Burien, WA). Blood samples were collected in heparinized BD Vacutainer tubes (BioVision, CA). Plasma was separated by centrifuging blood at 4 °C for 10 min at 1500 g. The samples were collected following a custom-designed protocol to ensure rigor and reproducibility. In addition, fresh blood samples were also obtained at the University of Washington. To evaluate the stability of the newly identified metabolites, blood samples were processed immediately or 24 hrs after the blood draw. The biospecimen collection protocol was approved by the IRB from the University of Washington.

### 2.3. Preparation of phosphate buffer

Buffer solution (100 mM) was prepared by dissolving 1124.0 mg anhydrous Na_2_HPO_4_ and 250.0 mg anhydrous NaH_2_PO_4_ in 100 g D_2_O. TSP was added to achieve a final concentration of 50 μM. The calculated pH of the buffer solution was 7.4 and the measured pH was 7.33. This buffer was used without further pH correction.

### 2.4. Solutions of standard compounds for spiking experiments

One mL stock solutions (1 and 50 mM) for all standard compounds were prepared, separately, by weighing the compounds and dissolving them in an appropriate amount of D_2_O. Further, using 1 mM solutions, 100 μM solutions of the standard compounds were prepared in phosphate buffer in D_2_O to obtain NMR spectra under conditions identical to those used for blood and plasma samples.

### 2.5. Blood and plasma metabolite extraction

Frozen samples were thawed at room temperature, homogenized, and 300 – 500 μL samples were mixed with methanol in a 1:2 ratio (v/v) (for plasma) or a mixture of methanol and chloroform in a 1:2:2 ratio (v/v/v) (for whole blood), vortexed, and incubated at −20 °C for 20 min. The mixtures were centrifuged at 13,400 g for 30 min to pellet proteins/cell debris. Supernatants (top aqueous layer for whole blood) were transferred to fresh vials and dried using a Vacufuge centrifuge concentrator (Eppendorf, Enfield, CT) or using nitrogen gas. The dried samples were mixed with 200 μL (for 3 mm NMR tube) or 600 μL (for 5 mm NMR tube) phosphate buffer in D_2_O containing 50 μM TSP and transferred to NMR tubes.

### 2.6. NMR spectroscopy

NMR experiments were performed at 298 K on a Bruker Avance III 800 MHz spectrometer equipped with a cryogenically cooled probe and Z-gradients suitable for inverse detection. A few spiking experiments were performed on a Bruker Avance III 600 MHz spectrometer with a wide bore magnet equipped with a room temperature probe and Z-gradients suitable for inverse detection. The one-pulse or 1D NOESY pulse sequence (for standard compounds) along with the CPMG (Carr-Purcell-Meiboom-Gill) pulse sequence (for whole blood and plasma samples), all with residual water signal suppression using presaturation, were used for ^1^H 1D NMR experiments. To confirm unknown metabolite identification, spectra were also obtained after each addition of 1–10 μL stock solution (1 or 50 mM) of the authentic compounds to the samples. Parameters used for the one pulse/1D NOESY experiments were, a 9615 Hz spectral width, 25 s recycle delay, 128 scans, and 32,768 time-domain points. Parameters used for the CPMG experiments were 9615 Hz spectral width, 5 s recycle delay, 256 scans, 32,768 time-domain points, and 256 ms CPMG pulse train length. The FIDs were Fourier transformed after zero filling by a factor of two and multiplied using an exponential window function with a line broadening (LB) of 0.3 or 0.5 Hz. In addition, to aid unknown metabolite identification, homonuclear two-dimensional (2D) experiments, such as ^1^H–^1^H double quantum filtered correlation spectroscopy (DQF-COSY) and ^1^H–^1^H total correlation spectroscopy (TOCSY) experiments, were performed for both whole blood and plasma samples (after metabolite extraction). The 2D experiments were performed with suppression of the residual water signal by presaturation during the relaxation delay. For DQF-COSY and TOCSY experiments, a sweep width of 9615 Hz was used in both dimensions; 512 FIDs were obtained with t_1_ increments, each with 2048 complex data points. The number of transients used was 16 and 40, and the relaxation delay was 2.0 s and 1.0 s for DQF-COSY and TOCSY, respectively. The resulting 2D data were zero-filled to 4096 points in the t_2_ dimension and 1024 points in the t_1_ dimension. A 90° shifted squared sine-bell window function was applied to both dimensions before Fourier transformation. Chemical shifts were referenced to the internal TSP signal for both 1D and 2D spectra. The Bruker Topspin version 4.1.4 or 3.6.5 software package was used for NMR data acquisition, processing, and analyses.

### 2.7. Peak assignment and unknown metabolite identification

Peak assignments for already identified metabolites relied on our prior publications on the blood metabolome [14–17]. Unknown metabolite identification involved a combination of literature/database searches [18, 19], comparison with chemical shift, peak multiplicity, and J couplings measurements from spectra of standard compounds obtained under conditions identical to the blood and plasma samples, and comprehensive analysis of 2D DQF-COSY and TOCSY spectra. The putative new compounds were subsequently confirmed by spiking with authentic compounds.

## 3. Results and discussion

**Fig. 1.**
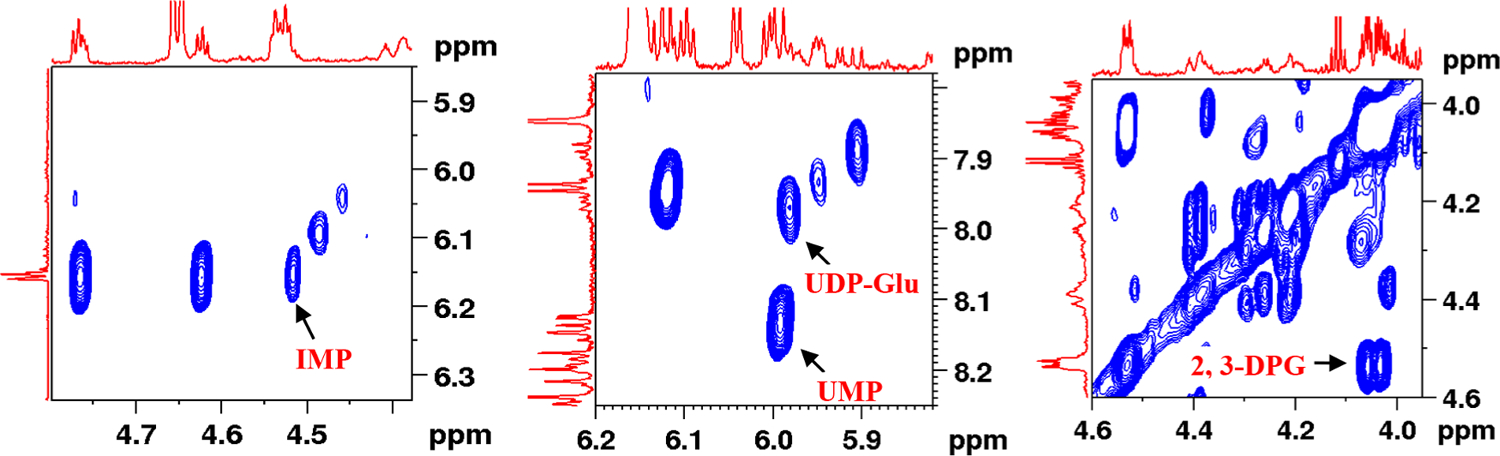
Portions of a typical 800 MHz (cryo-probe) 2D ^1^H–^1^H TOCSY spectrum of a human blood obtained after metabolite extraction. A few of the newly identified metabolites are highlighted: IMP: inosine monophosphate; UMP: uridine monophosphate; UDP-Glu: uridine diphosphate glucose; 2,3-DPG: 2,3-diphosphoglycerate. A 350 μL blood extract dissolved in 600 μL D_2_O buffer in a 5 mm NMR tube was used.

**Fig. 2.**
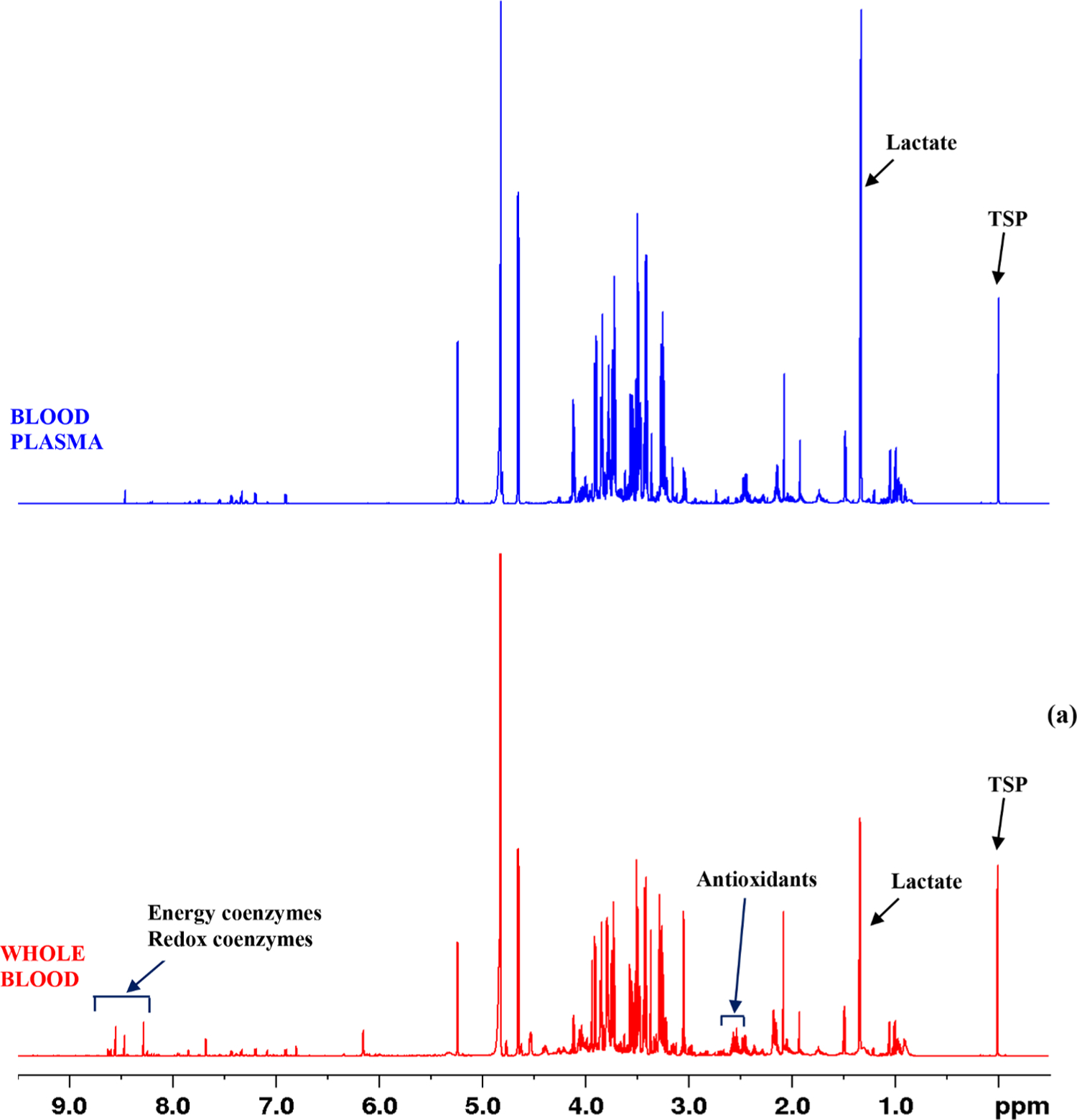

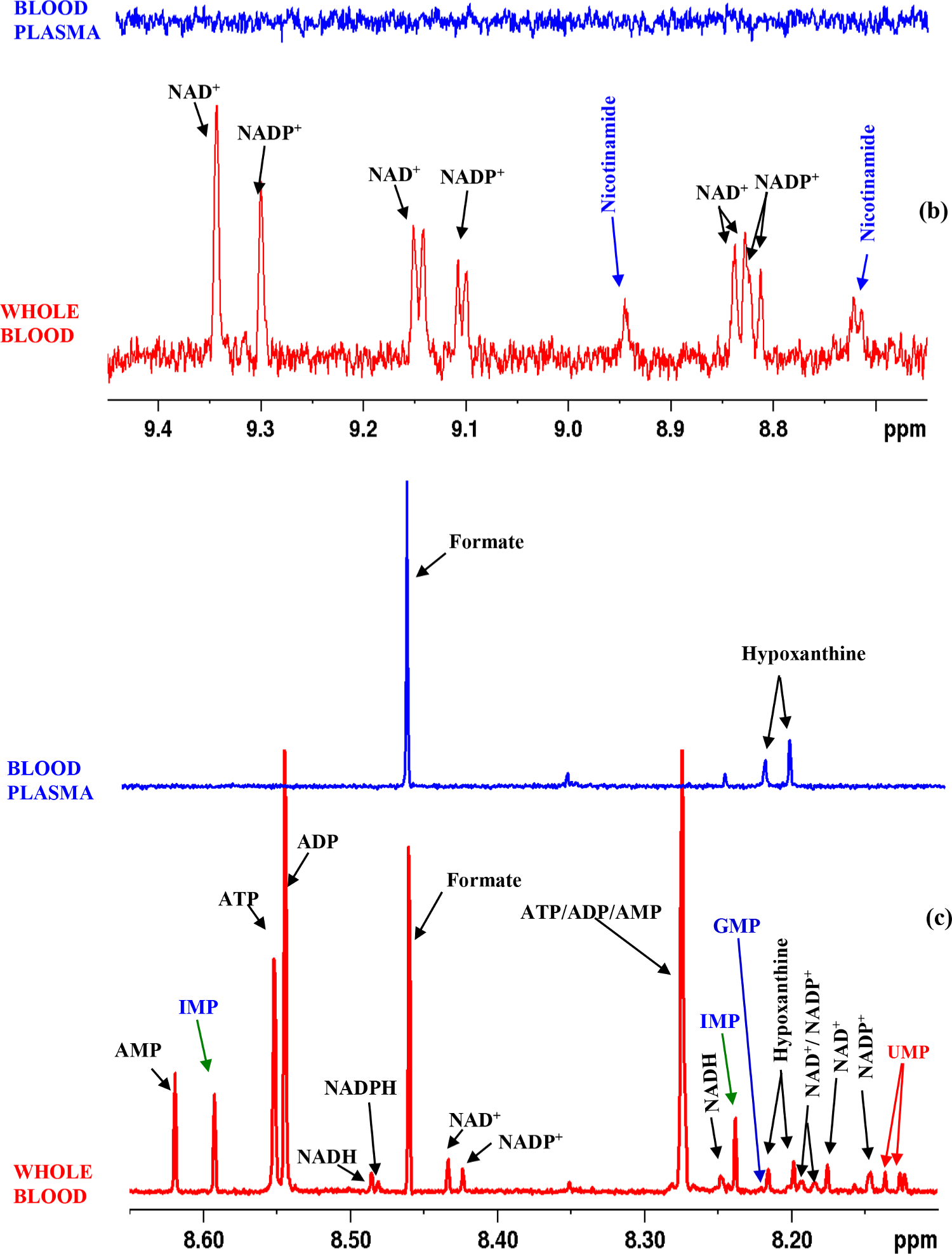

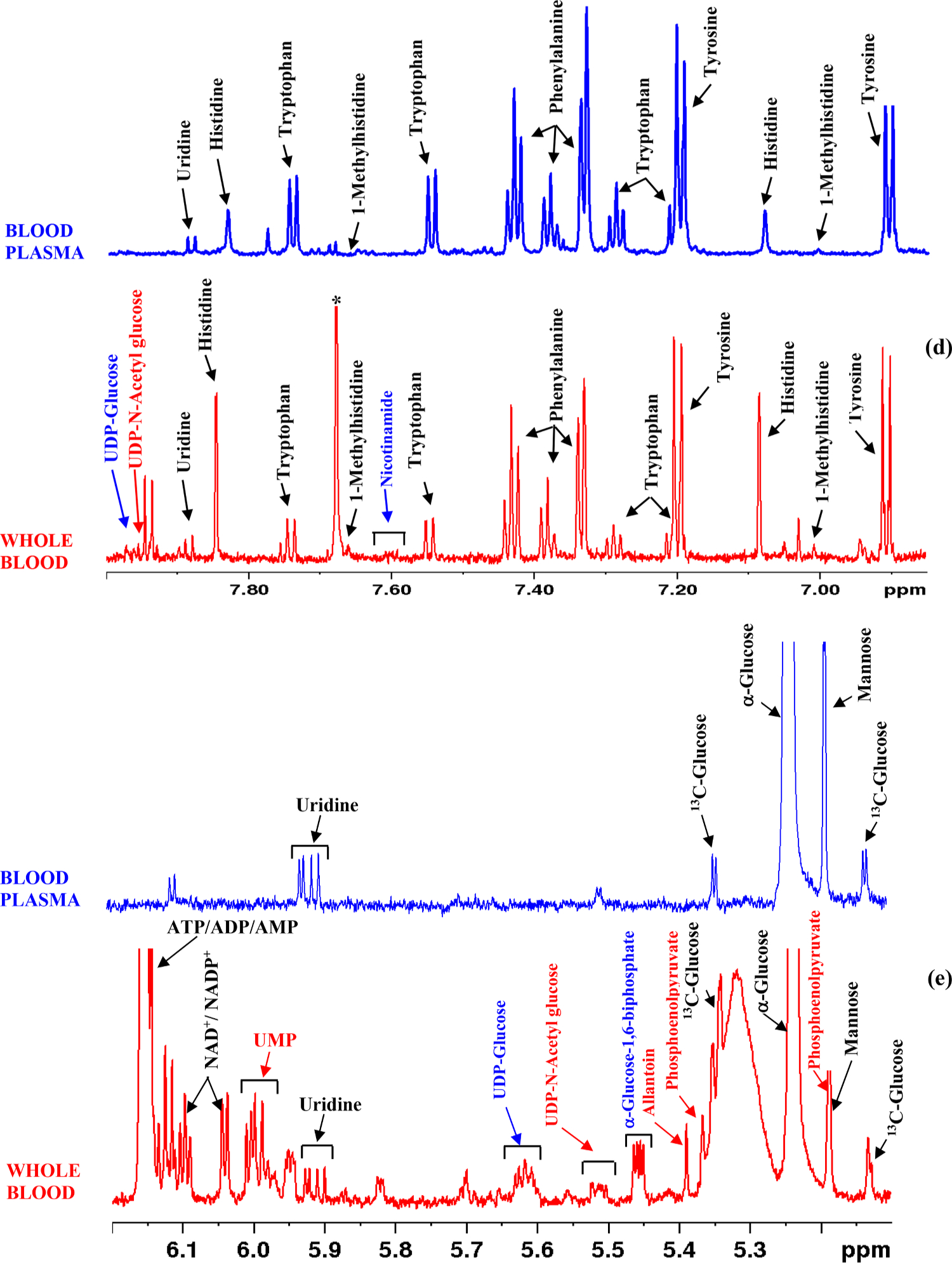

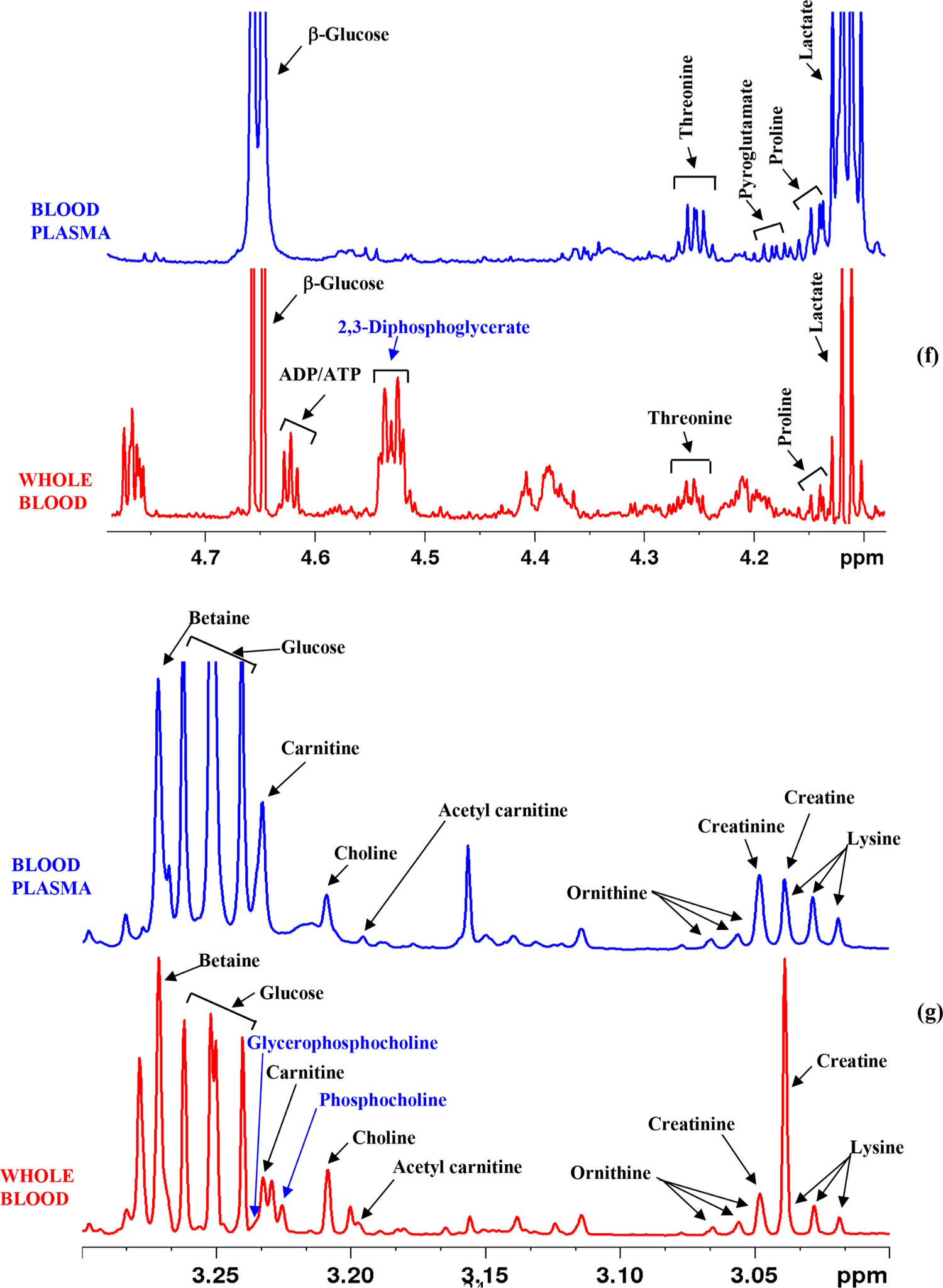
(a) Typical 800 MHz (cryo-probe) 1D CPMG ^1^H NMR spectra of whole blood (red) and blood plasma (blue) obtained after metabolite extraction and shown with expanded regions (b-g) with highlighting of the newly identified metabolites in red or blue, for clarity. NAD^+^: nicotinamide adenine dinucleotide (oxidized); NADH: nicotinamide adenine dinucleotide (reduced); NADP^+^: nicotinamide adenine dinucleotide phosphate (oxidized); NADPH: nicotinamide adenine dinucleotide phosphate (reduced); ATP: adenosine triphosphate; ADP: adenosine diphosphate; AMP: adenosine monophosphate; IMP: inosine monophosphate; UMP: uridine monophosphate; UDP: uridine diphosphate; GMP: guanine monophosphate. * Residual chloroform solvent peak. A 350 μL whole blood or plasma extract dissolved in 600 μL D_2_O buffer in a 5 mm NMR tube was used.

This study focused on expanding the identifiable metabolites in human blood using NMR spectroscopy. To achieve the goal, whole blood and blood plasma specimens were subjected to comprehensive analyses combining 1D and 2D NMR techniques, databases, spectra of authentic compounds obtained under conditions identical to blood and plasma, and spiking with authentic compounds. The study builds on the results from our earlier efforts to expand the identified blood metabolites [14–16] and expand the number of quantifiable blood metabolites by ~15%. The newly identified metabolites are UDP-N-acetyl glucose (UDP: uridine diphosphate), allantoin, 2,3-diphosphoglycerate (2, 3-DPG), α-d-glucose-1,6-biphosphate (G16BP), UDP-glucose (UDP-Glu), glycerophosphocholine (GPC), guanosine monophosphate (GMP), inosine monophosphate (IMP), nicotinamide, phosphocholine (PC), phosphoenolpyruvate (PEP), and uridine monophosphate (UMP) (Table 1). Fig. 1 shows regions from a typical 2D TOCSY NMR spectrum highlighting a few of the newly identified metabolites, 2,3-DPG, UDP-Glu, IMP, and UMP. Fig. 2 shows regions of the spectra for whole blood as well as blood plasma samples with peak annotations added for the newly identified metabolites. Results from the current study, in combination with our previously published results, now provide access to nearly 90 blood metabolites, which is the highest to date, and they all are quantifiable using a simple 1D NMR experiment.

Excluding exogenous compounds like supplements, drugs, or their metabolites, the NMR detected metabolite profiles of human blood or blood plasma are generally almost identical for different samples, at least qualitatively, because of the homeostatic regulation of blood, although the concentration of metabolites varies somewhat across subjects. Our strategy for unknown metabolite identification involved the following: (1) First, peaks in the 1D NMR spectrum that were unassigned to date were collected by comparing the NMR spectrum with spectra having annotations for identified metabolites as reported previously [14–16]; (2) The unassigned peaks were then tentatively assigned based on chemical shift databases, blood metabolite literature search, and their association with metabolic pathways [20]; (3) 2D DQF-COSY and TOCSY spectra were obtained and then comprehensively analyzed to identify peaks for the tentatively identified metabolites; (4) Blood spectra were compared with spectra for standard compounds of tentatively identified metabolites, obtained under identical conditions as for blood and plasma; and finally, (5) Spiking experiments using authentic compounds were used to validate the identified metabolites.

Generally, unknown metabolite identification is one of the major challenges in the metabolomics field. Specifically, for ^1^H NMR, the narrow dispersion of peaks, sensitivity of chemical shifts to sample conditions, broad concentration range of metabolites, and structural similarity for numerous metabolites pose challenges for unknown metabolite identification. Although databases and software tools are helpful for tentative identification, they are inadequate for unambiguous assignments. Hence, a one-time establishment of the identity for unassigned peaks using an actual blood NMR spectrum with peak annotations for the characteristic peaks is extremely useful. Due to the high similarity of blood spectra, peak assignments made using a single spectrum are applicable for any blood spectrum, when obtained under similar conditions.

All the newly identified metabolites were detected in whole blood, not in blood plasma (Fig. 2). Further, all these are phosphorus-containing metabolites except for allantoin and nicotinamide. It is well-known that human blood contains ~45% blood cells by volume. Since nearly 99% of blood cells are red blood cells (RBCs), new metabolites mainly arise from the RBCs. Interestingly, energy coenzymes (ATP, ADP, and AMP) and redox coenzymes (NAD^+^, NADH, NADP^+^, and NADPH) that we have previously identified in blood are also phosphorus-containing metabolites and similarly arise from the RBCs [16, 17].

There are some distinct advantages to measuring whole blood for metabolomics applications. Separating plasma from whole blood involves sample preprocessing, which potentially carries the risk of cellular metabolites seeping into the plasma due to hemolysis. Contamination of plasma with RBC metabolites due to hemolysis is common and plasma hemoglobin is a sensitive hemolysis marker [21]. Hence, varying levels of hemoglobin were found even in normal plasma and the hemoglobin correlated positively with many metabolites (including ATP) that are associated with hemolysis [22]. To address this issue, it has been suggested to exclude metabolites that are associated with hemolysis since it is difficult to exclude hemolyzed plasma samples from analysis [23]. However, the exclusion of metabolites is not practical since virtually all NMR-detected metabolites in plasma are common to blood cells [16]. Therefore, the use of whole blood not only avoids the confounding effects associated with hemolysis but also provides access to an expanded pool of metabolites that arise from both extracellular and intracellular matrices in one step with no additional efforts compared to plasma metabolomics. Hence, the identification of new metabolites in whole blood in this study represents a significant advancement in blood metabolomics.

**Fig. 3.**
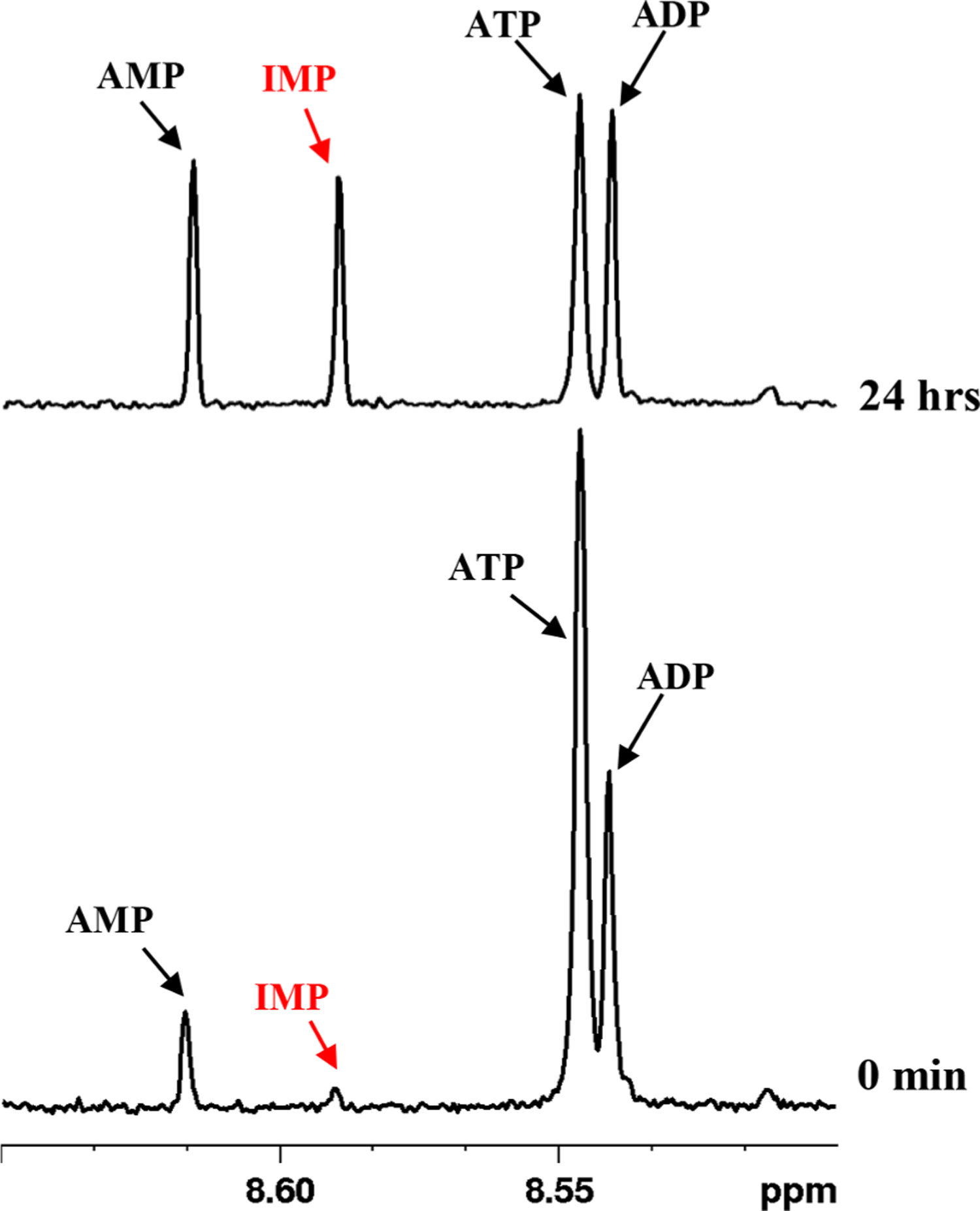
Portions of 800 MHz 1D CPMG ^1^H NMR spectra of whole blood extracted immediately (0 min) or 24 hrs after the blood draw. IMP peak intensity increases enormously in blood extracted 24 hrs after the blood draw with a concomitant decrease of ATP and an increase of ADP and AMP peaks.

Many metabolites are notoriously unstable, and their concentrations depend on biospecimen harvesting, metabolite extraction, and measurement conditions. In blood, we have investigated this instability for many important metabolites such as energy coenzymes, redox coenzymes, and antioxidants [16, 17, 24]. A lack of recognition of the unstable nature of such metabolites deleteriously affects inferences from blood metabolomics studies. In the current study, to identify unstable metabolites among the newly identified metabolites, we compared spectra of blood samples processed immediately or 24 hrs after the blood draw. The results show that the level of IMP increases enormously with a concomitant decrease of the ATP level; AMP and ADP also increase with time (Fig. 3). This is because ATP undergoes hydrolysis to form ADP, which in turn gets hydrolyzed to form AMP [25]. Further, AMP undergoes deamination to form IMP [25, 26]. These results indicate that in blood ATP is converted to IMP. Biologically, IMP is a precursor for AMP and GMP, and it plays an important role in intracellular purine metabolism [27]. In blood, IMP formed from ATP can outweigh its physiological concentration, which indicates the critical need to avoid or account for such preanalytical variations in blood metabolomics studies.

**Fig. 4.**
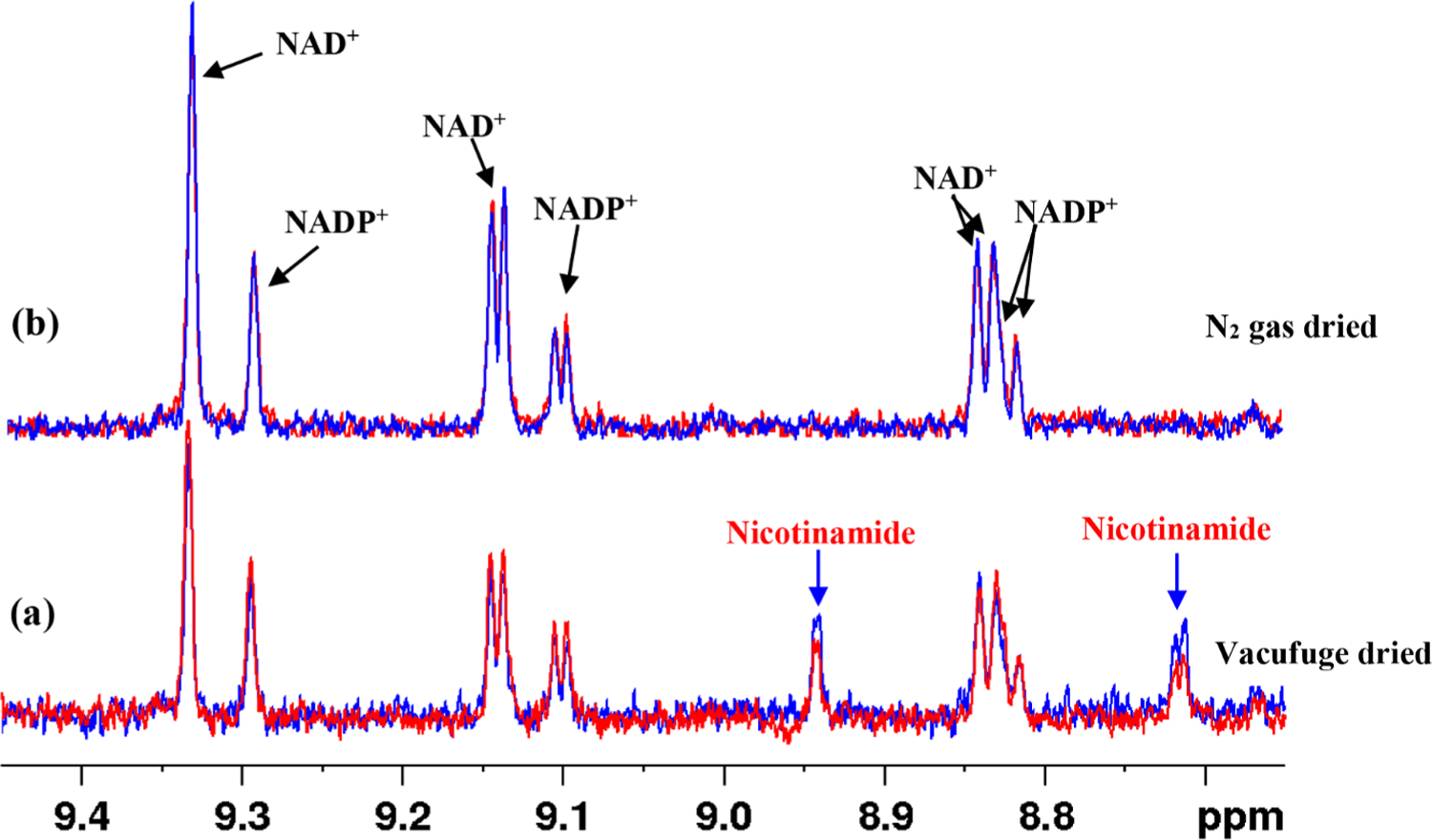
Portions of 800 MHz 1D CPMG ^1^H NMR spectra of two human blood samples processed using two different conditions: (a) blood samples were vacufuge-dried to remove solvent after metabolite extraction; (b) blood samples were dried by blowing nitrogen gas to remove solvent after metabolite extraction. In the vacufuge-dried samples, nicotinamide peaks appear with a concomitant decrease of NAD^+^ peaks (a).

Metabolically, nicotinamide is a major precursor to NAD^+^ synthesis [28]. We detected nicotinamide in blood samples processed using vacufuge drying whereas it was undetectably low in samples dried using nitrogen gas (Fig. 4). Further, the NAD^+^ peak intensity was reduced in the vacufuge-dried samples when compared to samples dried using nitrogen gas (Fig. 4). This result is likely because vacufuge drying generally causes a rise in the sample temperature, which results in the breakdown of NAD^+^ to nicotinamide. NAD^+^ is the most abundant redox coenzyme in blood as well as in other biological specimens and vacufuge drying is often utilized for metabolite extraction in the metabolomics field. Hence, it is important to be aware that such sample processing grossly confounds the physiological concentrations of nicotinamide as well as other labile NAD metabolites.

## 4. Conclusion

NMR spectroscopy is an important analytical platform for blood metabolomics because of its numerous unsurpassed characteristics that include its high reproducibility and quantitative capabilities. The relatively small number of metabolites identified by NMR compared to mass spectrometry has restricted the scope of applications. Ongoing efforts focused on improving metabolite identification include the development of chemical shift databases and software tools. Databases and software tools alone are inadequate for unambiguous unknown metabolite identification since chemical shifts are sensitive to numerous factors including solvent, pH, temperature, and sample concentration. Human blood is under strict homeostatic control and hence metabolite profiles from different blood samples are almost qualitatively identical. This is reflected in their NMR spectra, which invariably yield quasi-identical peak patterns, excluding exogenous metabolite signals. Using such spectra, a one-time establishment of peak identification provides a reliable template for routine applications. The current study used this strategy and took advantage of technological advances in NMR to comprehensively analyze blood spectra. In combination with our earlier studies, the results of the current study provide access to an unprecedented number of metabolites using a simple ^1^H 1D NMR. Such efforts are anticipated to further expand the limits of NMR- detectable blood metabolites in the near future.

Newly identified metabolites arise from blood cells, with a majority of them being phosphorus compounds. Many of the key metabolites we identified earlier in blood samples, including energy and redox coenzymes, which are fundamental to cellular functions, also arise from blood cells and are phosphorus-containing compounds. We have shown that many of these are quite labile, such that their concentrations depend on specimen collection, processing, and measurement conditions. It is important to take such metabolite instability into consideration for reliable interpretation of blood metabolite data. Overall, this study demonstrates that NMR-based metabolomics of whole blood offers access to a large and growing pool of metabolites with virtually no additional effort when compared to the conventionally used blood plasma or serum metabolomics.

## Figures and Tables

**Table 1:** Characteristics peaks for the blood metabolites newly identified by ^1^H NMR. ^1^H chemical shifts for authentic compounds obtained under conditions identical to blood are shown for comparison. Chemical shifts for isolated peaks in the human blood spectrum are shown in bold.

Identified Metabolite	Human blood	Authentic compound
Uridine diphosphate-N-acetylglucose (UDP-glucNAc)	**5.5131** (m); 7.965 (d)	2.0848 (s); 3.5604 (t); 3.816 (m); 3.8676 (d); 3.883 (d); 3.9274 (dd); 3.9402 (dd); 3.8864 (dt); 4.1944 (m); 4.2536 (m); 4.2962 (m); 4.3756 (m); 5.5219 (dd); 5.9759 (d; *J* = 8.130); 5.9913 (d; *J* = 4.721); 7.9652 (d; *J* = 8.130)
Allantoin	**5.3893** (s)	5.3910 (s)
2,3-diphosphoglycerate (2,3-DPG)	4.057 (m); 4.0335 (m); **4.5295** (m)	4.0289 (m); 4.0683 (m); 4.5382 (m)
α-d-glucose-1,6-biphosphate* (G16BP)	**5.4562** (dd)	1.1824 (m); 1.3462 (m); 1.6566 (m); 1.8055 (m); 1.9986 (br. m); 3.1548 (m); 3.5297 (m); 3.6402 (t); 3.7940 (t); 3.9456 (br. m); 4.1018 (m); 5.4568 (dd)
Uridine diphosphate-glucose (UDP-glu)	**5.6192** (m); 5.9822 (m); 7.966 (d)	3.4761 (t); 3.5451 (dt); 3.786 (m); 3.8569 (d); 3.8725 (d); 3.8979 (dd); 3.9107 (dd); 4.2096 (m); 4.2587 (m); 4.2994 (m); 4.3852 (m); 5.6093 (dd); 5.9926 (m); 7.9666 (d; *J* = 8.174)
Glycerophosphocholine (GPC)	**3.2345** (br. s)	3.2345 (s); 3.6892 (m); 4.3319 (m)
Guanosine monophosphate (GMP)	**8.2203** (s)	3.9954 (m); 4.3265 (m); 4.4917 (m); 5.9447 (d; *J* = 6.085); 8.2186 (s)
Inosine monophosphate (IMP)	4.5161 (m); 6.150 (d); 8.2379 (s); **8.5922** (s)	4.0226 (m); 4.376 (m); 4.5197(m); 6.1554 (d; *J* = 5.840); 8.2366 (s); 8.5913 (s)
Nicotinamide	7.6006 (dd); **8.7186** (m); **8.9447** (d)	7.5995 (dd); 8.2538 (m); 8.7171 (m); 8.9442 (d)
Phosphocholine (PC)	**3.2258** (s)	3.2257 (s); 3.597 (m); 4.1686 (m)
Phosphoenolpyruvate (PEP)	5.1882 (t); **5.3667** (t)	5.1896 (t); 5.3668 (t)
Uridine mono phosphate (UMP)	**5.9933** (d; *J* = 8.134); 6.007 (d; *J* = 5.389); **8.1306** (d; *J* = 8.135)	3.9741 (m); 4.0309 (m); 4.2657 (m); 4.3626 (t); 4.4302 (t); 5.9933 (d; *J* = 8.134); 6.0070 (d; *J* = 5.389); 8.1311 (d; *J* = 8.134)

Chemical shifts were measured (with an error of ± 0.001 ppm) with reference to the peak from the internal standard, TSP. J couplings are measured in Hz. Spectrum from 350 μL blood that was extracted, dried and dissolved in 600 μL D_2_O buffer and used for measuring chemical shifts in ppm. s, singlet; br. s, broad singlet; d, doublet; dd, doublet of doublets; t, triplet; dt, doublet of triplets; m, multiplet; br. m, broad multiplet.

* The standard compound used was α-D-glucose-1,6-biphosphate tetra(cyclohexyl ammonium) salt hydrate.

## Data Availability

Data will be made available on request.
